# Case Report of Ogilvie's Syndrome Following Emergency Haemostatic Subtotal Abdominal Hysterectomy at University Teaching Hospital of Butare, Rwanda

**DOI:** 10.24248/eahrj.v4i1.629

**Published:** 2020-06-26

**Authors:** Eugene Tuyishime, Marie Grace Mutuyimana, Evariste Zigiranyirazo, Theogene Twagirumugabe

**Affiliations:** a College of Medicine and Health Sciences, University of Rwanda; b Department of Anesthesia and Critical Care, University Teaching Hospital of Butare; c Department of Gynecology and Obstetrics, University Teaching Hospital of Butare

## Abstract

**Background:**

Acute colonic pseudo-obstruction or Ogilvie's syndrome is a rare condition that usually develops due to a dysregulated autonomic nervous system following a medical or surgical condition. With delayed diagnosis, it may lead to bowel ischemia and perforation with poor prognosis.

**Case:**

We report a case of a 33 years old female, Gravida 1, Para1, who developed severe abdominal distension following abdominal haemostatic hysterectomy due to a severe postpartum haemorrhage and shock requiring epinephrine infusion after a spontaneous vaginal delivery. The postpartum haemorrhage was due to both atony and posterior cervical tear. Two initial administrations of neostigmine 2 mg mixed with atropine 0.5 mg were unsuccessful, but an insertion of a flexible recto-sigmoid cannula allowed a slight decompression. A subsequent third dose of neostigmine 2 mg mixed with atropine 0.5 mg was followed with a remarkable flatus evacuation and complete decompression.

**Conclusion:**

Prompt diagnosis and management of Ogilvie's syndrome is crucial in order to avoid subsequent complications. In case of postoperative cecal and colonic distension without mechanical obstruction, Ogilvie's syndrome should be suspected as this will ensure timely and adequate management of patients at risk including obstetric patients.

## BACKGROUND

The first description of Ogilvie's syndrome was provided in 1948 by William Henry Ogilvie. The condition is characterized by signs and symptoms of a mechanical obstruction of the small or large bowel in the absence of a mechanical cause^1^. The true incidence is unknown, but it ranges between 9% and 19% among patients with risk factors such as severe infection, cardiac events, neurologic events, major surgery, and metabolic imbalance.^2,3,12^

The diagnosis of Ogilvie's syndrome is made based on signs of non-mechanical abdominal distension on a plain abdominal X-ray or CT scan^7^. The management depends on the severity of the syndrome and include supportive care, anticholinesterase medications, colonoscopy and surgery.^8,9,10^

In low resource settings without a readily available CT scan, it may be difficult to diagnose the syndrome. Suspicion of Ogilvie's syndrome based on the clinical presentation may avoid late diagnosis and subsequent complications. We report a case of Ogilvie's syndrome successfully managed in the Intensive Care Unit (ICU) at the University Teaching Hospital of Butare (UTHB), Rwanda.

### Case Presentation

A 33 years old female Gravida 1, Para 1, with a previous history of pelvic inflammatory disease but no abdominopelvic surgery, was referred to the Obstetric unit of UTHB from a 153 km-distant district hospital for severe postpartum haemorrhage. The patient had experienced normal spontaneous vaginal delivery. The UTHB has 500 beds and 6 operating rooms. It conducts approximately 8,000 surgeries each year.

Upon arrival at the hospital, she had received 2 litres of Intravenous (IV) normal saline 0.9% and Lactate Ringer, epinephrine infusion and transfused with 2 units of packed red blood cells. Initially, a diagnosis of uterine atony was made and a haemo-static B-Lynch procedure performed without success.

As she continued to bleed following subtotal haemostatic hysterectomy, a further clinical examination revealed a posterior cervico-uterine tear that was repaired successfully. She was transfused with additional 11 units of packed red blood cells, 7 fresh frozen plasma, 5 units of platelets and 3 units of cryo-precipitate.

Due to a persistent hemodynamic instability she was admitted to the ICU, and an infusion of epinephrine was maintained for 2 days. On the day 3 postICU admission, she developed a progressive abdominal distention while she was still under the instruction of not taking anything by mouth (*nihil per os).*

**FIGURE 1.**
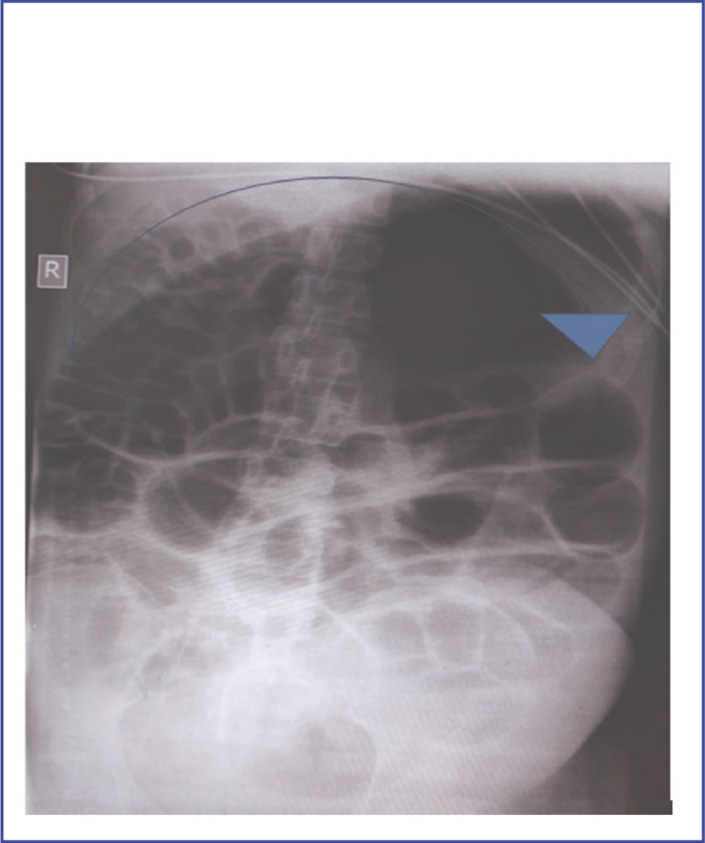
Plain abdomen radiography of the patient showing a dilatation of proximal part of the colon up to approximately the splenic flexure (Blue arrow).

A physical examination revealed an abdominal distension and tenderness on palpation, tympanic on percussion with decreased bowel sounds on auscultation. A plain abdominal X-ray showed dilated bowels (Figure 1).

A digital rectal exam did not reveal any faecaloma and a paralytic ileus was suspected. Results of laboratory investigations indicated that levels of Full Blood Count (FBC), electrolytes, lactate, urea, and creatinine were in normal ranges. The patient was managed by decreasing the frequency and dose of opioids and the insertion of the nasal gastric tube (NGT) for aspiration without improvement.

On day 5, the ICU treating team evoked the diagnosis of uncomplicated Ogilvie syndrome. A slow IV injection of neostigmine 2 mg mixed with atropine 0.5 mg was administered without improvement. A second same dose was given on the following day but patients showed no improvement. Endoscope specialist was contacted for decompressive colonoscopy but was not readily available. A tentative insertion of a recto-sigmoidal cannula with a flexible tube allowed a first flatus evacuation. A third dose of neostigmine 2 mg mixed with atropine 0.5 mg was administered and was followed by a significant evacuation of flatus allowing a decompression of the abdomen and relieve of symptoms. On day 6 of her admission in ICU, the patient was successfully extubated and weaned from ventilator. She was discharged from ICU on day 7 to the Obstetric Unit, and discharged from the hospital on day 11 post ICU discharge without any complication or reoccurrence of any sign of abdominal distention.

## DISCUSSION

Ogilvie Syndrome also known as Acute Colonic Pseudo Obstruction (ACPO) was first described as an acute colonic obstruction in absence of mechanical obstruction in 2 patients diagnosed with retroperitoneal tumours infiltrating the splanchnic and destroying the coeliac plexus^1^. The syndrome usually occurs in hospitalized patients and commonly associated various conditions (Table 1) such as severe illness, surgery, or as a complication of a metabolic imbalance especially hypokalaemia.^2,3^ The pathophysiology may involve an imbalance of the autonomic nervous system with an increased sympathetic tone over a decreased parasympathetic tone^4^. This results in a decreased motility of the proximal colon while distal colon whose para-sympathetic innervation depends on the sacral spinal segments is conserved, thus obstructing this distal colon. Proximal colon and caecum are then massively dilated to the extent that this exposes to a high risk of colonic ischemia and perforation if an urgent decompression is not performed. When the syndrome is complicated by a perforation, the resulting peritonitis is associated with a high rate of mortality that can reach 40% in high income countries and probably be much higher in resource limited settings in low income countries^5^.

**TABLE 1.** conditions commonly associated with Ogilvie's syndrome^2,3^

Trauma, especially fractures Obstetrical surgery, especially involving spinal anesthesia Pelvic, abdominal, or cardiothoracic surgery Major orthopedic surgery Severe medical illness, such as pneumonia, myocardial infarction, or heart failure Neurologic conditions Chemotherapy (eg, all-trans retinoic acid, methotrexate, vincristine) Retroperitoneal pathology, such as malignancy or hemorrhage One of the above plus metabolic imbalance or medication administration (eg, narcotics, phenothiazine, calcium channel blockers, alpha-2-adrenergic agonists, epidural analgesics)

Complications are more likely to occur in case of delayed recognition generally after 6 days from initial symptoms and in case of high tension with a colonic dilatation exceeding 9-12 cm of the diameter of the lumen^6^.

Different predisposing factors are reported but obstetric, gynaecologic and pelvic surgeries are among the most important factors occurring in 20% of reported cases of Ogilvie syndrome^6^. Early diagnosis within 48 hours and a successful management achieved within a short time frame may play a significant role in avoiding any severe complication. The diagnosis is generally made upon signs and symptoms of abdominal distension with a plain abdominal X-ray displaying dilatation of the proximal colon without any distinguishable mechanical obstruction on CT-Scan when it is performed^7^. When there is a colonic perforation commonly complicating the syndrome, signs of peritonitis are remarked and a pneumoperitoneum is seen on both abdominal X-ray and CT-Scan.

Once recognized, the initial management of an uncomplicated Ogilvie's syndrome includes ruling out of predisposing factors like opioids, and a supportive management by giving IV fluids for resuscitation, correction of potential electrolytes imbalance and insertion of a nasogastric tube for gastric emptying.

Anti-cholinesterase medications such as neostigmine and pyridostigmine, by the increase of the acetylcholine and its effects on promoting colonic motor activity are also used in non-complicated cases. Neostigmine in particular, has successfully been used in more than 80% of cases^8^. The optimal dose is estimated to 2-2.5 mg administered as slow IV in a setting with close cardiac monitoring. Atropine should be available as bradycardia generally complicates this administration of neostigmine and may lead to an asystole among other complications^8^.

In some cases, neostigmine may cause colonic perforation due to resulting hyperperistalsis in the already largely dilated colon. However, perforation occurs when management of Ogilvie syndrome is delayed and particularly when administration of neostigmine is not attempted. Generally 2-3 doses are recommended^8^.

In case of neostigmine failure, alternative treatment is decompressive colonoscopy with or without insertion of decompressive colorectal tube. In up to 90% of cases, decompression occur as a result of colonoscopy^9^.

Surgical approach is only indicated in case of failure of those conservative approaches and in case of severe and ischemic colon and perforation.^1,10^ Perendoscopic caecostomy has also been successfully used when conservative management failed but is indicated in the absence of ischemic colon and perforation. However, this technique may be associated with a high morbidity^10^. Finally, given the pathophysiology of this syndrome, a thoracic epidural analgesia owing to its inhibition of sympathetic effects, has also been successfully used in some cases of Ogilvie syndrome^11^.

## CONCLUSION

Prompt diagnosis and management of Ogilvie's syndrome is crucial in order to avoid subsequent complications. In case of postoperative cecal and colonic distension without mechanical obstruction, Ogilvie's syndrome should be suspected as this will ensure timely and adequate management of patients at risk including obstetric patients.

## List of Abbreviations

ICU: : Intensive Care Unit
UTHB: : University Teaching Hospital of Butare
IV: : Intravenous
FBC: : Full Blood Count
NGT: : Nasal Gastric Tube
ACPO: : Acute Colonic Pseudo-Obstruction
